# Oleuropein Decreases Cyclooxygenase-2 and Interleukin-17 Expression and Attenuates Inflammatory Damage in Colonic Samples from Ulcerative Colitis Patients

**DOI:** 10.3390/nu9040391

**Published:** 2017-04-15

**Authors:** Tiziana Larussa, Manuela Oliverio, Evelina Suraci, Marta Greco, Roberta Placida, Serena Gervasi, Raffaella Marasco, Maria Imeneo, Donatella Paolino, Luigi Tucci, Elio Gulletta, Massimo Fresta, Antonio Procopio, Francesco Luzza

**Affiliations:** 1 Department of Health Sciences, University of Catanzaro “Magna Graecia”, 88100 Catanzaro, Italy; tiziana.larussa@gmail.com (T.L.); m.oliverio@unicz.it (M.O.); e.suraci@libero.it (E.S.); marta.greco@unicz.it (M.G.); robplac91@gmail.com (R.P.); serena.gervasi@yahoo.it (S.G.); raffaellamarasco1@gmail.com (R.M.); graziaimeneo@hotmail.it (M.I.); paolino@unicz.it (D.P.); gulletta@unicz.it (E.G.); fresta@unicz.it (M.F.); procopio@unicz.it (A.P.); 2 Pathology Unit, Pugliese-Ciaccio Hospital, 88100 Catanzaro, Italy; gigitucci@libero.it

**Keywords:** oleuropein, ulcerative colitis, cyclooxygenase-2, interleukin-17, olive oil

## Abstract

Oleuropein (OLE) is the major phenolic secoiridoid of olive tree leaves, and its antioxidant and anti-inflammatory activities have been demonstrated in in vitro and in vivo animal models. The aim of this study was to investigate the activity of OLE in the colonic mucosa from patients with ulcerative colitis (UC). Biopsies obtained during colonoscopy from 14 patients with active UC were immediately placed in an organ culture chamber and challenged with lipopolysaccharide from *Escherichia coli* (EC-LPS) at 1 μg/mL in the presence or absence of 3 mM OLE. The expression of cyclooxygenase (COX)-2 and interleukin (IL)-17 was assessed in total protein extracts from treated colonic biopsies by Western blotting. Levels of IL-17 were also measured in culture supernatant by ELISA. A microscopic evaluation of the cultured biopsies was performed by conventional histology and immunohistochemistry. The expression of COX-2 and IL-17 were significantly lower in samples treated with OLE + EC-LPS compared with those treated with EC-LPS alone (0.80 ± 0.15 arbitrary units (a.u.) vs. 1.06 ± 0.19 a.u., *p* = 0.003, and 0.71 ± 0.08 a.u. vs. 1.26 ± 0.42 a.u., *p* = 0.03, respectively) as were the levels of IL-17 in culture supernatants of OLE + EC-LPS treated colonic samples (21.16 ± 8.64 pg/mL vs. 40.67 ± 9.24 pg/mL, *p* = 0.01). Histologically, OLE-treated colonic samples showed an amelioration of inflammatory damage with reduced infiltration of CD3, CD4, and CD20 cells, while CD68 numbers increased. The anti-inflammatory activity of OLE was demonstrated in colonic biopsies from UC patients. These new data support a potential role of OLE in the treatment of UC.

## 1. Introduction

Ulcerative colitis (UC) is a chronic bowel disease characterized by colonic mucosal inflammation and associated with immune system dysregulation [1]. Despite promising advances in the knowledge of the genetic, immune and inflammatory mechanisms, and potential environmental factors contributing to the disease, the pathogenetic scenario is still not fully understood [2]. UC therapy, whose goal is to induce and maintain remission, is presently based on anti-inflammatory nonsteroid drugs such as mesalamine, corticosteroids, and immuno-suppressive molecules [3]. With the advent of the biologic era over the last decade, drugs such as infliximab and other anti-tumor necrosis factor monoclonal antibodies have revolutionized the treatment strategy of inflammatory bowel disease (IBD), and novel biologic agents are still emerging. Nevertheless, these drugs are much more expensive than conventional therapy [4]. Furthermore, up to 20–30% of patients fail to respond to these drugs and concerns also arise about their potential adverse side effects [5]. Surgery is the last option in critical patients, but is often associated with short- and long-term complications [6]. Therefore, alternatives for a safer, cheaper, and more efficacious approach in managing UC patients are being sought.

Nutraceutical compounds, such as bioactive peptides, phytochemicals, and omega 3-polyunsaturated fatty acids, are currently under investigation for their potentially useful activities in IBD [7]. Dietary polyphenols are able to modulate intestinal inflammation, reducing oxidative stress and inflammation via several pathways [8]. Beneficial effects on human health from the oil, fruits, and leaves of the olive tree have been documented and attributed to their phenolic content [9]. Oleuropein (OLE), the major phenolic secoiridoid from the olive tree leaves, has been found to exert antioxidant, antiangiogenic and anti-inflammatory effects [10]. Observations made in animal models and healthy humans have shown the intraluminal stability of OLE in the small intestinal mucosa and the rapid degradation by the colonic microflora into its active metabolite hydroxytyrosol (HT) [11,12]. No adverse effects were reported from acute toxicity studies conducted in animal species and from human studies, supporting the safety profile of OLE and its metabolites [13,14].

In a mouse model of dextran sodium sulfate (DSS)-induced colitis, OLE reduced neutrophil, macrophage, and eosinophil accumulation in colon tissue and inhibited COX-2 expression, which is known to be enhanced in the colonic epithelium of UC patients [15]. In the same animal model, OLE was found to downregulate the Th17 response, a key proinflammatory pathway activated in IBD [16]. These findings led us to hypothesize that OLE may have the potential to reverse chronic inflammation in UC.

## 2. Materials and Methods

### 2.1. Patients and Sampling

Fourteen outpatients (8 males, 6 females, median age 59 years, range 39–80) with UC who underwent routine colonoscopy were enrolled in this study. Diagnosis of IBD was assessed in accordance with current clinical guidelines and criteria based on endoscopic, radiological and histopathologic examination [17]. Since the purpose of the study was to examine the anti-inflammatory effects of OLE in UC, we selected patients who had mild to moderate active ulcerative colitis, defined by a Mayo score ranging from 4 to 6, median 6 [18]. Eight UC patients had pancolitis, the remaining 6 had left-sided colitis. All the patients were treated with oral and topical mesalamine at the time of biopsy, were not on immunomodulating medications including thiopurines or biologics and had suspended any steroid treatment, both oral and local, at least three months earlier. None of them had ever been treated with anti-TNF alpha agents, cyclosporine, or methotrexate. Characteristics of the patients are summarized in Table 1. During colonoscopy, ten biopsies were taken from endoscopically affected areas but avoiding frankly ulcerated areas. All biopsies were taken with the same biopsy forceps and by the same endoscopist (FL) to ensure the samples were uniform in size.

### 2.2. Ex Vivo Organ Culture

Biopsy samples were immediately placed on steel grids in the central well of an organ culture dish containing RPMI 1640, 5% fetal bovine serum, 10 mmol/L l-glutamine, 0.25 μg/mL amphotericin B (all obtained from Invitrogen, Carlsbad, CA, USA). Then, the dishes were placed in an organ culture chamber at 37 °C in 95% O_2_, 5% CO_2_ for 4 and 20 h, and biopsies were stimulated or not (untreated controls) with LPS from *Escherichia coli* (EC-LPS, *Escherichia coli* Serotype O127:B8 Lipopolysaccharide, Sigma-Aldrich, Milan, Italy) at a concentration of 1 μg/mL, to induce an inflammatory response, in the absence or presence of OLE at a final concentration of 3 mM. OLE was obtained using a novel sustainable synthetic strategy, as described by Procopio et al. [19]. Due to its high water solubility, OLE was dissolved in the same medium used for organ culture, to give a stock solution at a concentration of 20 mM. One biopsy specimen from each treatment was collected after 4 h and embedded in paraffin for histologic evaluation, while the remaining samples were snap-frozen after 20 h and then stored at −80 °C for further protein extraction. To exclude direct toxicity of OLE, the viability of the biopsies over the culture period was checked by hematoxylin/eosin (H & E) staining of frozen tissue sections. The explants were considered viable only if the morphology of the tissue was intact with well-defined crypts, epithelial surface and adequate and strong uptake of H & E.

Preliminary experiments in our Laboratory were performed to optimize the dose range (0.1, 0.5, 1, 3, 5, and 10 mM of OLE) and time course (6, 8, 12, 16, and 20 h).

### 2.3. Extraction of Total Proteins

Total proteins were extracted from biopsies after 20 h of culture, using lysis buffer (50 mmol/L HEPES pH 7.6, 150 mmol/L NaCl, 1% Triton X-100, 1 mmol/L Na_3_VO_4_, 10 mmol/L NaF, 30 mmol/L Na_4_P_2_O_7_, 10% glycerol, 1 mmol/L benzamidine, 1 mmol/L dithiothreitol (DTT), 10 lg/mL leupeptin, and 1 mmol/L phenylmethylsulfonyl fluoride (PMSF)) (all obtained from Sigma-Aldrich S.r.l., St. Louis, MO, USA). After incubation for 30 min in ice, the membranes were centrifuged for 30 min at 3100 *g* at 4 °C. The supernatant containing the total proteins was retained, the protein concentration was determined by the Bradford method to ensure equal protein loading prior to Western blot analysis, and aliquots were stored at −80 °C.

### 2.4. Western Blot Analysis

Levels of COX-2 and IL-17 were assessed in total protein extracts from cultured biopsy specimens after 20 h. Total proteins (40 µg) were resolved by sodium dodecyl sulfate-polyacrylamide gel electrophoresis and electrophoretically transferred onto an Immobilon-P membrane (Amersham, Life Sciences Inc., Buckinghamshire, UK); a 10% gel was used in the detection of COX-2 whilst a 12% was used in the detection of IL-17. Ponceau S staining was performed to confirm the equal loading and transfer of proteins. The membranes were blocked for 1 h in ‘blocking buffer’ (5% nonfat dry milk in 10 mmol/L Tris-HCl, 100 mmol/L NaCl, and 0.1% Tween 20, pH 7.6). This was followed by incubation with COX-2 and IL-17 mAbs (Santa Cruz Biotechnology, Santa Cruz, CA, USA), diluted 1/500 in blocking buffer overnight at 4 °C, and then with horseradish peroxide-conjugated goat anti-rabbit IgG mAb, diluted 1/2000 for 1 h (Santa Cruz Biotechnology). All the blots were stripped and reprobed with an anti-β-actin antibody diluted 1/5000 (from Sigma), and the secondary antibody used was horseradish peroxide-conjugated goat anti-mouse IgG mAb (Santa Cruz Biotechnology) diluted 1/2000. Chemiluminescence luminol reagent (Santa Cruz Biotechnology) was used for detection. Bands were measured densitometrically, and their relative density calculated based on the density of the β-actin bands in each sample. Values were expressed as arbitrary densitometric units (a.u.) corresponding to signal intensity.

### 2.5. Measurement of IL-17 in the Culture Supernatants

Culture supernatants were collected after 20 h and stored at −80 °C. Levels of IL-17 were measured by enzyme-linked immunosorbent assay (ELISA, R & D Systems, Minneapolis, MN, USA) following the manufacturers’ instructions. The amount of cytokine was quantified within each supernatant in duplicate. Results were given as pg/mL, normalized on protein content and the mean value with standard deviation was calculated.

### 2.6. Histology

Preliminary data experiments in our Laboratory suggested an optimal 4 h culture in order to minimise any alteration in gland cytoarchitecture upon microscopic evaluation. Biopsy specimens were fixed in 4% paraformaldehyde. Following dehydration and paraffin-embedding, biopsies were cut into 3–10 μm thick sections and stained with H & E. Colonic sections were processed through deparaffinization, rehydration using Dako En Vision Detection™ FLEX+, Mouse, High pH system (Peroxidase/DAB+, K5007, Dako Italia SRL, Milan, Italy), and immunohistochemical staining was performed using a panel of rabbit monoclonal antibodies against CD3-CD4-CD20-CD68 at 1:200 dilution (Novus Biological, Milan, Italy). Antigen expression and distribution was visualized using Dako Autostainer after 60 min at room temperature. Hematoxylin was used for counterstaining. All the specimens fulfilled the criteria of intact morphology and H & E uptake in order to be included in this final analysis. Conventional histology and immunohistochemistry were performed by an expert pathologist (LT) who was blinded to the clinical and experimental data. For quantification, a blinded scoring was used and rated with no (−), few (+), moderate (++) and intense (+++) staining.

### 2.7. Statistical Analysis

Mann-Whitney U test and the paired two tailed Student’s test was used for statistical analyses, as appropriate. A *p*-value of less than 0.05 was considered statistically significant.

### 2.8. Ethical Considerations

All the patients gave informed consent to the study and for the procurement of further biopsies in addition to those needed for clinical management during colonoscopy. The study was approved by the local Research Ethics Committee (n. 2013.31).

## 3. Results

### 3.1. OLE Decreases COX-2 Expression in Colonic Biopsy Culture

**Figure 1:**
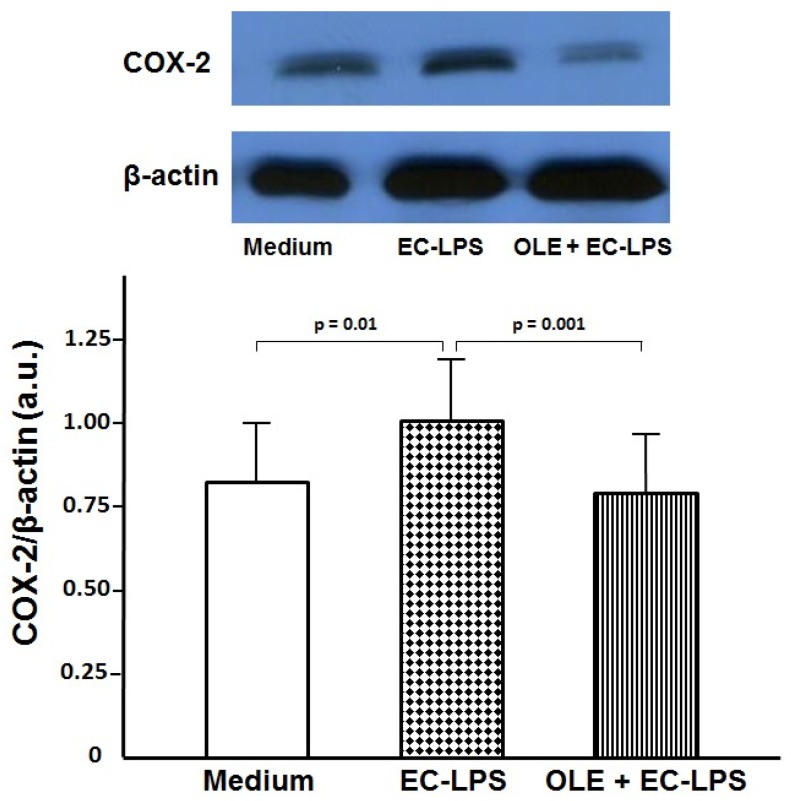
Oleuropein (OLE) decreases cyclooxygenase (COX)-2 expression in colonic biopsy culture. Levels of COX-2 observed by Western blotting of total protein extracts from colonic biopsiesof patientswith ulcerative colitis (*n* = 14) treated with medium (untreated samples) or lipopolysaccharide from *Escherichia coli* (EC-LPS) at 1 µg/mL, in the absence or presence of 3 mM OLE for 20 h in an organ culture chamber. β-actin was used as loading control. Values are expressed as mean values ± SD of arbitrary units (a.u.). The immunoblot panel is one representative experiment.

Using a tissue culture model for biopsies of human colonic mucosa, we showed that OLE prevents LPS-induced increase of COX-2 in LPS-treated colonic mucosa from UC patients (Figure 1). EC-LPS challenge led to an increase in COX-2 levels at 20 h of incubation in UC colonic mucosa compared with untreated samples (1.06 ± 0.19 a.u. vs. 0.84 ± 0.16 a.u., *p* = 0.01), thus confirming this as a good model of inflammation. Moreover, in cultured biopsies treated with EC-LPS + OLE, COX-2 production was highly reduced compared with LPS treatment alone (0.80 ± 0.15 a.u. vs. 1.06 ± 0.19 a.u., *p* = 0.001, Figure 1).

### 3.2. OLE Suppresses IL-17 in Both Mucosa and Supernatant of Colonic Biopsy Culture

**Figure 2:**
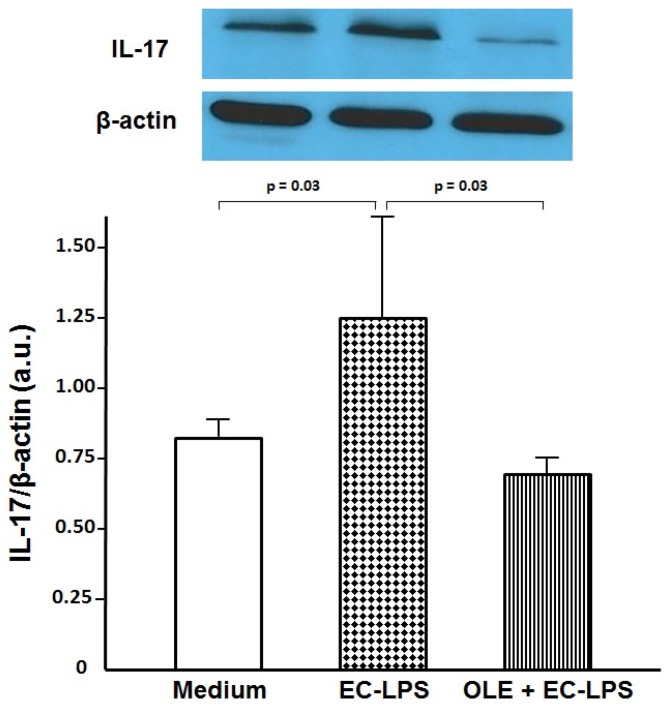
Oleuropein (OLE) decreases interleukin (IL)-17 expression in colonic biopsy culture. Levels of IL-17 observed by Western blotting of total protein extract from colonic biopsies of patients with ulcerative colitis (*n* = 14) treated for 20 h in an organ culture chamber with medium (untreated samples) or lipopolysaccharide from *Escherichia coli* (EC-LPS) at 1 µg/mL, in the absence or presence of 3 mM OLE. β-actin was used as loading control. Values are expressed as mean values ± SD of arbitrary units (a.u.). The immunoblot panel is one representative experiment.

**Figure 3:**
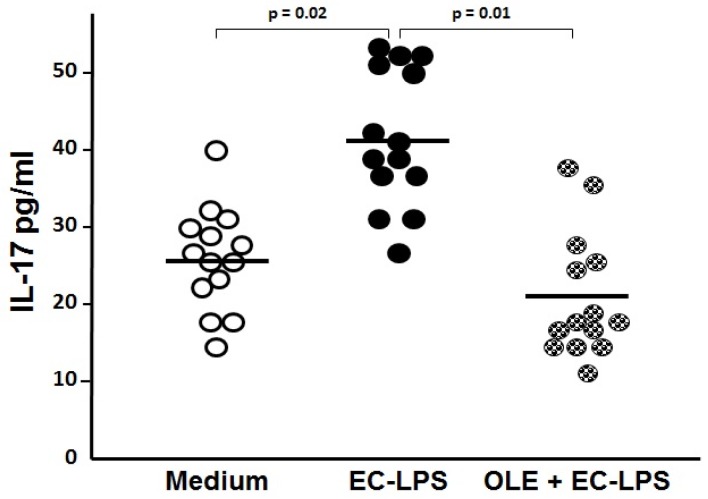
Oleuropein (OLE) decreases interleukin (IL)-17 levels in supernatant of colonic biopsy culture. Levels of IL-17 by ELISA observed in supernatant from colonic biopsies of patients with ulcerative colitis (*n* = 14) treated for 20 h in an organ culture chamber with medium (untreated samples) or lipopolysaccharide from *Escherichia coli* (EC-LPS) at 1 µg/mL, in the absence or presence of 3 mM OLE. Values are given in pg/mL as scattered plots with mean values.

To assess whether OLE was able to switch off the mucosal inflammation in human EC-LPS stimulated colonic mucosa, we analyzed the expression of the pro-inflammatory cytokine IL-17 in colonic tissue and its release in the supernatant from cultured biopsies (Figure 2 and Figure 3). An increased expression of IL-17, along with its high secretion, was observed in samples following stimulation with EC-LPS compared with the non-stimulated samples (1.26 ± 0.42 a.u vs. 0.80 ± 0.09 a.u., *p* = 0.03 and 40.67 ± 9.24 pg/mL vs. 25.9 ± 6.92 pg/mL, *p* = 0.02, respectively, Figure 2 and Figure 3). The enhanced production of IL-17 in EC-LPS stimulated samples was efficiently lowered with the addition of OLE, in both mucosa and culture supernatant (0.71 ± 0.08 a.u. vs. 1.26 ± 0.42 a.u., *p* = 0.03, and 21.16 ± 8.64 pg/mL vs. 40.67 ± 9.24 pg/mL, *p* = 0.01, respectively, Figure 2 and Figure 3).

### 3.3. OLE Attenuates Inflammatory Damage in Colonic Biopsy Samples

**Figure 4:**
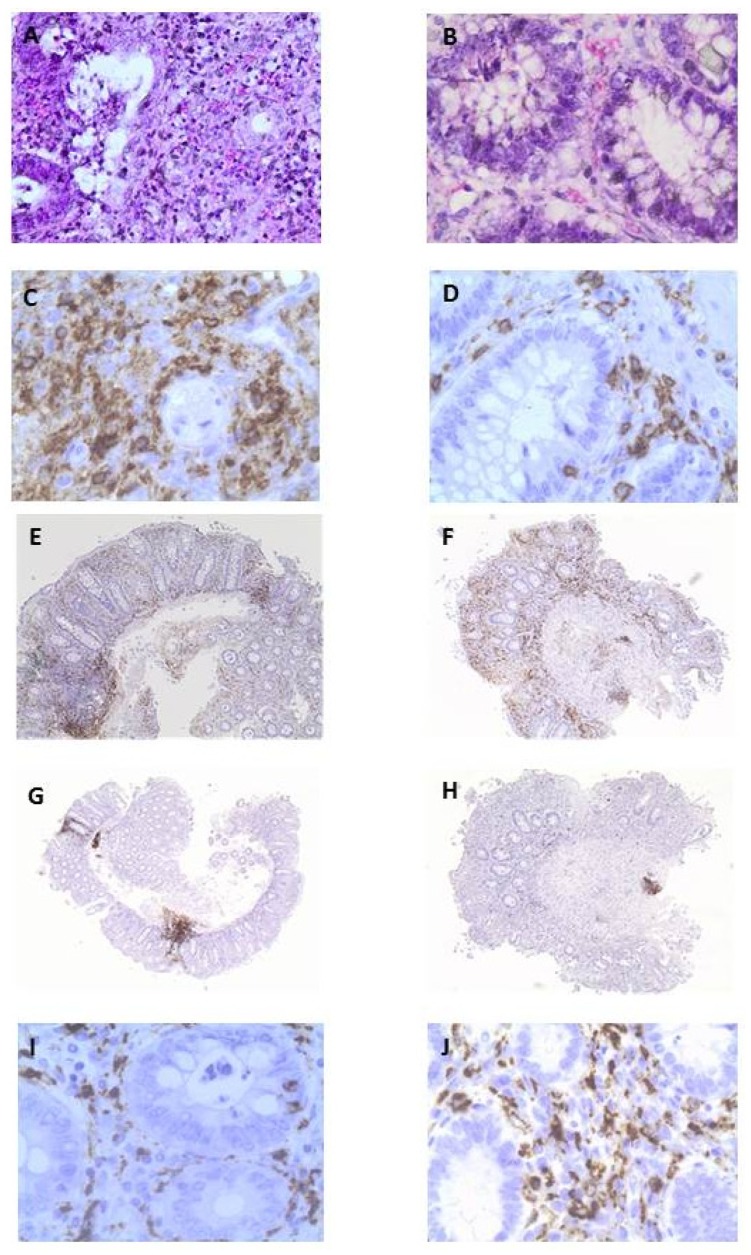
Anti-inflammatory effects of oleuropein (OLE) in colonic biopsies from patients with ulcerative colitis. Representative images of tissue sections of OLE-treated and untreated samples from the same patient. (**A**) Dense inflammatory infiltrate of leukocytes in the lamina propria, with epithelial damage, necrosis of the surface cells, and loss of mucin secretion (score +++; H & E stain, 400× magnification) in a biopsy specimen treated with lipopolysaccharide from *Escherichia coli* (EC-LPS) alone; (**B**) Decreased infiltration of leukocytes, mainly mononuclear cells, preservation of mucin secretion and presence of goblet cells in the superficial portion of the glands (score +; H & E stain, 400× magnification) in a biopsy specimen treated with OLE + EC-LPS; (**C**) A greater number of intra-epithelial and sub-mucosal T lymphocytes infiltration (score +++; immunohistochemical stain for CD3, 400× magnification) in a biopsy specimen treated with EC-LPS alone; (**D**) Decreased T lymphocytosis in the submucosa and lamina propria, and preservation of mucin secretion (score +; immunohistochemical stain for CD3, 400× magnification) in a biopsy specimen treated with OLE + EC-LPS; (**E**) Enhanced infiltration of intraepithelial and sub-mucosal T helper lymphocytes in a biopsy specimen treated with EC-LPS alone (score +++; immunohistochemical stain for CD4, 40× magnification); (**F**) Decreased infiltration of intraepithelial and sub-mucosal T helper lymphocytes in a biopsy specimen treated with OLE + EC-LPS (score +; immunohistochemical stain for CD4, 40× magnification); (**G**) A greater number of B lymphocytes are observed as the dominant cell types in the aggregates of biopsies treated with EC-LPS alone (score ++; immunohistochemical stain for CD20, 200× magnification); (**H**) The aggregates are significantly reduced in size and are confined in the submucosa as inflammatory residuals in biopsy specimens treated with OLE + EC-LPS (score +; immunohistochemical stain for CD20, 200× magnification); (**I**) Histiocytic infiltration consistent with the degree of the inflammation in the submucosa of the biopsy specimens treated with EC-LPS (score +; immunohistochemical stain for CD68, 400× magnification); (**J**) Increased histiocytic infiltration along with restoration of mucin secretion in biopsy specimens treated with OLE + EC-LPS (score ++; immunohistochemical stain for CD68, 400× magnification).

EC-LPS challenged samples showed a dense inflammatory infiltrate of leukocytes in the lamina propria, with epithelial damage, necrosis of the surface cells, and loss of mucin secretion (Figure 4A). A decreased infiltration of leukocytes, mainly mononuclear cells, with preservation of mucin secretion and the presence of goblet cells in the superficial portion of the glands was found in biopsy specimens treated with OLE + EC-LPS (Figure 4B). Using immunohistochemistry, a greater number of intra-epithelial and sub-mucosal T lymphocytes (Figure 4C) and T helper lymphocytes (Figure 4E) was observed in biopsy specimens treated with EC-LPS alone compared with that found in biopsy specimens treated with the addition of OLE (Figure 4D,F). Similarly, the greater number of B lymphocytes, as the dominant cell types in the aggregates in biopsy specimens treated with EC-LPS alone (Figure 4G), were significantly reduced and confined in the submucosa in biopsy specimens treated with the addition of OLE (Figure 4H). Conversely, and in attempting to repair, the histiocytic infiltrate significantly increased in biopsy specimens treated with OLE (Figure 4J) compared with that found in biopsy specimens treated with EC-LPS alone (Figure 4I).

## 4. Discussion

In this study, we demonstrated that OLE exerts broad anti-inflammatory actions in inflamed colonic tissue from UC patients. Little information is available on the benefits of OLE, the main olive oil secoiridoid phenol, in human health [20]. Data from animal models indicate that OLE attenuates the inflammatory process in DSS-induced colitis by downregulating the expression of COX-2 and pro-inflammatory cytokines, such as TNF-alpha, IL-1β, IL-6 and IL-17 [15,21,22]. Nevertheless, there are no studies on the anti-inflammatory activity of OLE in patients with UC. With this in mind, OLE was used to treat colonic biopsies taken from UC patients with active disease in an organ-culture system.

The role of COX-2 in mediating the barrier dysfunction that contributes to colonic inflammation has been demonstrated in mice as has its overexpression in the colonic epithelium of UC patients [23,24]. As the key enzyme regulating the production of prostaglandins (PG), COX-2 is considered a central mediator of the inflammation process. Indeed, the therapeutic action of 5-aminosalicylic acid in treatment of UC patients relies on its inhibition of COX-2 activation [25,26]. Since we found that the expression of COX-2 was significantly reduced in OLE-treated colonic samples, this suggests that the OLE-induced anti-inflammatory effects in colonic tissue from UC patients could be mediated, at least in part, by the inhibition of COX-2 activity.

Inflammatory mediators such as the nuclear factor kappa-light-chain enhancer of activated B cells (NF-κB), TNF-α, and IL-1β have been demonstrated to enhance COX-2 expression [27,28,29]. Since data have been produced to show that OLE may significantly inhibit the activation of NF-κB along with the production of TNF-α and IL-1β [30,31], it may be argued that, in our setting, the OLE-induced inhibition of COX-2 activity may be mediated through the suppression of these factors.

Experiments of this study were also focused on IL-17, the key cytokine of the Th17 response which mediates pro-inflammatory functions such as the recruitment of neutrophils and the secretion of metalloproteinases [32]. IL-17 expression has been found to be increased in the colonic mucosa and serum of UC patients and this has been associated with the unbalanced immune response and the related inflammatory process which take place in the colonic mucosa [33,34]. In both protein extracts and supernatants from colonic biopsies taken from UC patients which were challenged with OLE, we found a significant decrease in IL-17 levels. Since the production of IL-17 has been associated with the activation of NF-κB [35], it could be hypothesized that OLE-induced inhibition of NF-κB is one of the mechanisms responsible for decreasing levels of IL-17 in our experiments.

In order to verify whether the OLE-induced decrease in expression of COX-2 and IL-17 was associated with an amelioration of the inflammatory process, we analyzed the samples at a microscopic level and using conventional histology and immunohistochemistry.

UC tissue usually exhibits a microscopic pattern of chronic active colitis, diffuse and uniform in distribution, which combines the presence of active inflammation and the features of chronic mucosal injury. Activity is defined as the presence of neutrophil-mediated epithelial injury, which may take the form of neutrophils infiltrating the crypt epithelium (cryptitis), gathering of neutrophils within crypt lumens (crypt abscesses), or by infiltration of surface epithelium with or without mucosal ulceration. Chronicity is defined by crypt architectural distortion, mainly represented by shortening of the crypts, and basal lymphoplasmacytosis [36]. Histologic remission is crucial in UC since prolonged inflammation promotes cellular damage and an uncontrolled regeneration process, which could lead to errors in DNA duplication and genetic mutation supporting cancer development [37]. Interestingly, treatment of biopsy samples with OLE led to an almost complete disappearance of the microscopic features of UC with a prominent decrease in the inflammatory infiltrate, absence of focal cryptitis/crypt abscesses and restoration of mucin-forming goblet cells.

The CD3+ and CD4+ subset of T lymphocyte cells are key players in UC tissue, promoting inflammation and tissue destruction; this is confirmed by their enhanced activation and decreased apoptosis leading to an increased infiltration which has been documented in the colonic mucosa of UC patients [38,39]. B lymphocytes also play an important role in the antibody-induced inflammatory process which takes place in this setting [40]. Using immunohistochemistry, we demonstrated a dramatic reduction in all of these cells, which was responsible for the overall attenuation of the inflammatory damage in OLE-treated colonic samples from UC patients.

The expression of CD68 is a marker of tissue macrophages/monocytes (histiocytes) in the colon [41]. Comparative analysis showed that the number of histiocytes in the mucosa of newly diagnosed and chronic UC patients increases with the chronicity and is not related to the severity of damage [42]. Since in UC tissue histiocytes represent a reaction to crypt rupture with extravasation of mucin, the increased infiltration of CD68+ cells we detected by immunohistochemistry in OLE-treated colonic samples may well be interpreted as an attempt to repair by those cells enhanced by the presence of OLE.

The data of this study confirm and extend previous in vitro and in vivo animal model observations regarding the anti-inflammatory effects of OLE. Nonetheless, up to now, no study has specifically dealt with the human gastrointestinal mucosa. The mechanisms by which OLE decreases levels of COX-2 and IL-17 in colonic tissue have not yet been elucidated, but this is beyond the scope of the study. Indeed, the entire modulatory process of inflammation in the organ culture model we used is more complex than that occurring in in vitro experiments, but is better in representing the intricate environment that would be found in an in vivo setting. OLE is generally the most prominent phenolic compound in olive cultivars and can reach concentrations of up to 140 mg g^−1^ on a dry matter basis in young olives and 60–90 mg g^−1^ of dry matter in the leaves [43]. The powder preparation of OLE which has been used here contains a very high proportion (>95%) of the active compound [19]. Many other commercially available preparations contain far less OLE, therefore presumably would have a lower anti-inflammatory effect. Furthermore, regarding the concentration used, it would be difficult to directly extrapolate from the ex vivo results obtained in this study to what may occur in vivo in the human colon. The EU Register of nutrition and health claims made on foods stated that olive oil polyphenols contribute to the protection of blood lipids from oxidative stress. For olive oil, which contains at least five milligrams of hydroxytyrosol and its derivatives (e.g., oleuropein complex and tyrosol) per 20 g, the beneficial effect is obtained with a daily intake of 20 g of olive oil [44]. Nonetheless, ad hoc designed pharmacodynamic and pharmacokinetic studies would elucidate these aspects.

Collectively, the findings of this study suggest that OLE has the potential to be used as a therapeutic agent in patients with UC.

## Figures and Tables

**Table 1:** Demographic and clinical characteristics of the 14 patients with ulcerative colitis who underwent biopsy sampling during colonoscopy.

Characteristic	Values
Sex	8 males; 6 females
Age (years)	59 (range 39–80)
Mayo Score	6 (range 4–6)
Pancolitis	8
Left-sided colitis	6
Oral/Topic Mesalamine	14
Previous azathioprine/anti-TNF alpha	-
Current oral/topic Steroids	-

Values are numbers or median with range, as indicated.
